# Laparoscopic Sleeve Gastrectomy: To Suture or not to Suture Staple Line?

**DOI:** 10.7759/cureus.2992

**Published:** 2018-07-17

**Authors:** Ghulam Siddiq, Waqas Aziz, Samina Khizar, Mohammad Ijlal Haider, Aneela Razzaq, Zahid Ahmad, Mahum Nadeem, Chaudhary Muhammad Junaid Nazar

**Affiliations:** 1 General Surgery, Shifa international hospital , Islamabad, PAK; 2 General Surgery, Shifa International Hospital, Islamabad, PAK; 3 General Surgery, Shifa Internationa Hospital, Islamabad, PAK; 4 Surgery, Shifa International Hospital, Islamabad, PAK; 5 Nephrology, Shifa International Hospital, Islamabad, PAK; 6 Internal Medicine, Sharif Medical and Dental College, Lahore, PAK; 7 Nephrology and Renal Transplantation, Shifa International Hospital, Islamabad, PAK

**Keywords:** obesity, laparoscopic, sleeve gastrectomy, leak, staple line, bleeding

## Abstract

Objective

To assess the outcome and safety of staple line over-sewing for patients undergoing laparoscopic sleeve gastrectomy (LSG).

Study design and location

Retrospective descriptive analysis conducted at Shifa International Hospital Islamabad.

Materials and methods

Consecutive patients undergoing LSG as a treatment for morbid obesity from October 2013 to December 2016 were included in the study after approval from the ethical review board. Patients were divided into two groups: group A who underwent reinforcement using Vicryl 2.0 and group B where no reinforcement was done.

Results

A total of 225 patients underwent LSG between October 2013 and December 2016, including 147 females (65.4%) and 78 males (34.6%). Both groups were comparable in terms of age, body mass index (BMI) and gender distribution (p-value more than 0.05). There was one leak in group A (1.36%), none in group B. The bleeding rate was 4.3% in group A and 2.7% in group B.

Conclusion

This was a retrospective analysis of all the patients who underwent LSG, and it was observed that there was no added benefit of sewing the staple line in terms of rate of bleeding and leak.

## Introduction

Over the last two decades, the role of bariatric surgery in the management of obesity and its related comorbidities and their complications has become well established. In the field of bariatric surgery, the procedures are still evolving and there is a constant struggle to improve patient outcome in terms of weight loss and achieving better control of comorbidities. Bariatric surgery improves the control of diabetes in nearly 90% of patients by lowering blood sugars, reducing the dosage of medication required for glycemic control achieving a remission rate of 78% [1, 2]. With the rising rates of obesity along with its comorbidities and its burden on health systems in both western world as well as developing countries, the need for better weight control and better treatment strategies is stronger than ever; it has been found to cost US$117 annually in the USA alone [3, 4].

The bariatric procedures can be as simple as gastric banding or more advanced and complicated techniques, such as biliopancreatic diversion or ileal transposition. More recently Roux-en-Y gastric bypass (RYGB) and sleeve gastrectomy (SG) have gained popularity because of their better results in terms of weight loss and type 2 diabetes mellitus (T2DM) control [5]. A recent meta-analysis conducted by Parikh et al. found the mean expected weight loss (EWL) at one year post laparoscopic sleeve gastrectomy (LSG) to be 56.7% and 70.1% at three years following LSG [6]. The technical simplicity along with smaller mean operative time, the absence of enteric anastomosis and less complication rate are all the reasons for the popularity of LSG, and reliable data is available on the positive effect of overall survival and quality of life of morbidly obese patients [7]. The learning curve for LSG is also shorter as compared to gastric bypass and other procedures which makes it easier for young surgeons to master the technique [8].

Sleeve gastrectomy is generally considered less invasive than RYGB surgery [9]. However, the risk of staple line leak and bleeding still remains one of its challenging complications. Leak rate has been reported in a retrospective review of 4888 patients to be 7% with the mean risk being 2.6% [10]. Despite the fact that there are a large number of studies assessing various methods of making the staple line secure, there is till date no consensus on which technique is best for reducing the risk of leak. These intra-operative techniques include over-sewing the staple line, using absorbable or non-absorbable suturing material, omental wrap, and fibrin glues and so forth [11].

Although only few centers perform these procedures, there has been an increase in overall trend towards bariatric surgery with better hospitals, expertise and improved post-operative care. There is no local data available on the rate of staple line leak. The aim of this study was to assess the outcome of our patients who underwent LSG and compare the rate of leak in those cases where the staple line was sutured versus those in which it was left as it is.

## Materials and methods

After the approval from the ethical review board, a retrospective analysis was conducted at Shifa international hospital Islamabad. It included the patients who underwent LSG from October 2013 to December 2016. The demographic data including age, gender, body mass index (BMI), comorbid conditions was collected on a self-designed performa from well-maintained medical records, and the operative details were extracted from the operative notes. The patients were then divided into two groups: Group A where the staple line was reinforced using sutures and group B where it was not reinforced. Both groups were assessed in terms of post-operative hospital stay and complications such as leak and bleeding from the staple line. The operative technique is explained below.

Operative technique

The patient was positioned on Allen stirrups and sequential compression devices were applied along with appropriate preoperative antibiotics and 60 mg of subcutaneous low-molecular-weight heparin. A five-port access technique was used as illustrated. The peritoneum was insufflated through a supra-umbilical incision 10 cm from the xiphoid process; insufflation was done up to 17 mmHg using a Veress needle technique. Next, a 10 mm camera port was placed and general laparoscopy was carried out using a 30 degree angled laparoscope. This was followed by two 12 mm working ports placed in the same transverse plane above the camera port; one 2 cm to the right of the midline and the other in the left midclavicular line. Both of these ports were used for dissection and stapling. A smaller 5 mm port was placed along the left subcostal margin lateral to the 12 mm port for retraction of the omentum and fundus of the stomach. Finally, a 5 mm infra-xiphoid epigastric port was placed for retraction of the left lobe of the liver by a self-retaining metallic Nathanson retractor. Following the establishment of the ports, the left lobe of the liver was retracted and the whole stomach was visualized. Once the pylorus was identified a window was created between the greater curvature of the stomach and the greater omentum to obtain access to the lesser sac. Using this window the entire greater curvature was devascularized starting 4 cm proximal to the pylorus and moving up to the esophagogastric junction at angle of His using a ligature. Particular care was taken at this stage due to the proximity of the spleen. In addition, the posterior wall of the stomach was also released from the underlying tissues with a combination of sharp and blunt dissection. With the completion of preparatory dissection, progress was made to the actual stapling stage. A 36 French bougie was inserted under laryngoscopic guidance down to the antrum of the stomach. A linear cutting endostapler is then used to staple and divide the stomach serially starting 4 cm from the pylorus and progressing up to the esophagogastric junction using the bougie as a guide to prevent deviation of the staple line. In some cases, this step was further reinforced by over-sewing the staple line with Vicryl 2/0 using an intracorporeal technique followed by a methylene blue leak test through a nasogastric tube used to replace the bougie. The transected gastric component was extracted through the left flank 12 mm port and sent for histopathological analysis. The final stage of the procedure was securing hemostasis and placing an 18 French drain along the staple line and securing the drain with silk 1. The ports were then extracted after desufflation of the peritoneum, local anesthetic was infiltrated along the port lines and the skin was closed using surgical staples followed by application of sterile dressing.

Postoperatively patients were started on sips of water on post-operative day one. On day two, they were progressed to oral rehydration salts (ORS). And from day three, they were started on a customized blenderized diet designed by the team nutritionist until eventual progression to solids. Regular post-operative gastrografin leak test was not part of the routine investigations. Postoperative leak and bleeding was assessed clinically in terms of tachypnea, tachycardia, out of proportion pain and increased analgesia requirement.

## Results

A total of 225 patients from October 2013 to December 2016 underwent the LSG procedure, including 147 females (65.4%) and 78 males (34.6%). Table 1 shows the demographic data of all the patients in both groups. Both groups were comparable in terms of age, BMI and gender distribution (p-value more than 0.05).

**Table 1:** Demographic data of two groups. BMI: Body mass index.

Group A: Staple Line Reinforced			Group B: Staple Line Not Reinforced		p-value
	Male	Female	Male	Female	
Gender %	42 (36.5%)	73 (63.5%)	36 (32.8%)	74 (67.2%)	0.125
Mean Age	38.8 (SD ± 11.78)	36.5 (SD ± 10.85)	38.9 (SD ± 12.50)	37.8 (SD ± 10.50)	0.684
BMI kg/m^2 ^	41.2 (SD ± 6.5)	47.2 (SD ± 7.5)	40.5 (SD ± 8.6)	45.2 (SD ± 6.7)	0.197
Total	115 (51.1%)		110 (48.9%)		

Table 2 shows the data on leak and bleeding cases in the two groups. There was only one leak which occurred in group A where the staple line was reinforced using Vicryl 2.0. The leak was picked up preoperatively during the methylene test and it was secured. There was no leak in group B as shown in Table 2. There were total eight cases (Group A- 5; Group B- 3) in which bleeding from the staple line occurred.

**Table 2:** Leak and bleeding in the two groups. BMI: Body mass index.

Group A: Staple Line Reinforced			Group B: Staple Line Not Reinforced		Total
	Male	Female	Male	Female	
BMI kg/m^2 ^	41.2 (SD ± 6.5)	47.2 (SD ±​​​​​​​ 7.5)	40.5 (SD ±​​​​​​​ 8.6)	45.2 (SD ±​​​​​​​ 6.7)	
Leak	0	1 (1.36%)	0	0	1
Bleeding	1 (2.38%)	4 (5.48%)	1 (2.77%)	2 (2.70%)	8

Out of the eight cases in which bleeding occurred one was identified intra-operatively and hemostasis was secured using liga-clips, seven cases needed to be taken back in the operating room within 24 hours because of fresh blood found in the drains. All seven cases underwent laparoscopy and bleeding from the staple line was identified and was secured using the liga-clips without any complications. These patients later had uneventful hospital stay and recovery. There was no mortality in our study groups; the only morbidity was associated with the one case where staple line leak occurred. On follow-up, there were no complications, no active complaints documented.

The patient in whom the leak had occurred was a young female, with no comorbidities. The leak was initially picked up intraoperatively and was secured. On post-op day two, there was froth seen in the drain, following which gastrografin follow through was done which revealed a small leak. We opted for the endoscopy and attempted to reseal the leak. The patient remained on total parenteral nutrition for about two weeks.

## Discussion

Sleeve gastrectomy over the years has become one of the most common bariatric procedure with its durable effects on maintaining weight and control of other comorbidities especially diabetes [12]. Although the debate still remains whether LSG is better than gastric bypass or not, the main challenge for the surgeons during the procedure is the leak and bleeding from the staple line [13]. In this single centre study, we compared the reinforcement of the staple line using vicryl 2.0 in LSG as opposed to leaving the staple line as it is (no reinforcement). And we found that there was no statistically significant difference between the two groups in terms of staple line leak and intra-operative and post-operative bleeding. The one leak that occurred in our group A was during the initial periods of the learning curve, and therefore we believe that it was more of a technical error during staple firing rather than the issue of reinforcement.

The results of our study differ from those which have been conducted in other parts of the world. In a systematic review, Gagner and Buchwald compared four different methods for securing the staple line. The different methods included no reinforcement, over-sewing using absorbable suture, bovine pericardium strips and absorbable membrane. There was 2.60% leak rate in no reinforcement group as compared to 2.04% and 3.30% in bovine pericardium strips and over-sewing, respectively. The lowest rate was observed in cases where the absorbable membrane was used [7]. In another prospective study, Rogula et al. compared over-sewing with no reinforcement; it was found that there was an increased risk of leak in no reinforcement group and suture group [14, 15]. This study, however, carried out on the resected gastrectomy specimens and does not truly depict the actual pressure in the live stomach. Also, in the presence of ischemia, the risk of leak would be higher whether the staple line is sutured or left as it is. In the present study, we found no statistically significant difference between both the groups in terms of leak. However, the operative time was significantly longer for patients where over-sewing was done, especially during the initial period of the learning curve. It decreased and stabilized over time and there was no time difference after 60 to 70 cases. This observation was similar to various other authors [13, 16].

In a randomized comparison, Shah et al. found no significant difference in the leak rates in their set of patients. In the subset of patients with BMI < 43 they found increased bleeding risk from the staple line in the group where it was not reinforced as compared to those where PSD (Peri-strips dry) were used [11]. In a review by Chen et al., the authors comment that there is no reason to believe that the reinforcement leads to the reduction in the rate of staple line leak or bleeding [17]. Contradictory results have been reported by Stamou et al. [18] and Karakoyun et al. [19]. However, Sajid et al. [20] in their prospective comparison concluded that there was no added advantage of reinforcement of the staple lines.

The current management of the staple line or anastomotic leaks is a step-wise approach. Non-operative and less invasive measures, such as percutaneous drainage, endoscopic repair of leak, and stent, are feasible, safe, and effective for staple line leaks in patients undergoing LSG [21, 22]. In the present study, the same approach was applied and the patient recovered in about six weeks of time post-operatively with no residual complications.

## Conclusions

This was a retrospective analysis of all the patients who underwent LSG, and it was observed that there was no added benefit of over-sewing the staple line in terms of rate of bleeding and leak. Results from other studies show a very controversial picture; therefore, prospective trials without any confounding factors are needed.
